# Complete Genome Sequences of Two *Escherichia coli* O157:H7 Phages Effective in Limiting Contamination of Food Products

**DOI:** 10.1128/genomeA.00519-14

**Published:** 2014-09-11

**Authors:** Yingying Hong, Yanying Pan, Nicholas J. Harman, Paul D. Ebner

**Affiliations:** Department of Animal Sciences, Purdue University, West Lafayette, Indiana, USA

## Abstract

We previously demonstrated that application of bacteriophages significantly reduced *Escherichia coli* O157:H7 contamination in spinach and ground beef. Here, we present the genomic sequences of two bacteriophages, vB_EcoS_FFH_1, a T5-like phage, and vB_EcoM_FFH_2, an rV5-like phage, used in those treatments.

## GENOME ANNOUNCEMENT

*Escherichia coli* O157:H7 is a shiga toxin-producing food-borne pathogen that results in over 60,000 illnesses each year in the United States alone (1). We have employed bacteriophages to limit *Salmonella* transmission in swine (2) and recently demonstrated that application of lytic bacteriophages to ground beef and spinach significantly reduced *E. coli* O157:H7 contamination (3). We selected two *E. coli* phages (vB_EcoS_FFH_1 [siphovirus] and vB_EcoM_FFH_2 [myovirus]) for genomic sequencing based on their broad spectrum and lytic capacity.

Phage DNA was purified from polyethylene glycol (PEG)-precipitated lysates and sequenced via pyrosequencing (454; Eurofins MWG Operon, Huntsville, AL) and sequences were assembled *de novo* using Newbler (version 2.6). Coding DNA sequences (CDSs) were predicted using Glimmer 3.0 (4) and annotation was performed using BLASTp for homology searching in the non-redundant protein sequences database in GenBank (5). tRNA genes were predicted using both tRNAscan-SE 1.21 (6) and ARAGORN (7). Terminal redundant ends (vB_EcoM_FFH_2) were identified using Tandem Repeat Finder (8).

The genome of vB_EcoS_FFH_1 has a length of 108,483 bp and a G+C content of 39.24%. Whole genome alignment revealed that vB_EcoS_FFH_1 showed 87% homology to T5 (GenBank accession no. AY543070) and therefore was classified as a T5-like phage. A total of 160 CDSs and 24 tRNA genes were predicted. Similar high numbers of tRNA genes are found in T5. Of the CDSs, 52 matched proteins with known functions, while 96 encoded previously identified hypothetical proteins. Twelve CDSs did not match any proteins in the NCBI non-redundant protein database. We identified putative *Rz* and *Rz1* genes based on Summer et al. (9). Highly similar sequences are also present in T5, but are not annotated in the three GenBank T5 complete genomes and other available T5-like phage genomes. One section (79,918 to 84,241) of the vB_EcoS_FFH_1 genome appeared largely absent from the three GenBank annotated T5 genomes, but present in the bV_EcoS_AKFV33 genome (another T5-like phage). Two putative tail fiber proteins and one hypothetical protein were identified in this section.

The genome of vB_EcoM_FFH_2 has a length of 139,020 bp and a G+C content of 43.61%. Whole-genome alignment revealed that vB_EcoM_FFH_2 shared 93% nucleotide homology to *E. coli* phage rV5 (GenBank accession no. DQ832317) indicating that vB_EcoM_FFH_2 is an rV5-like phage. A total of 220 CDSs and 6 tRNA genes were predicted. Of the CDSs, 57 matched proteins with known functions, while 156 matched previously identified hypothetical proteins. Seven CDSs were not homologous to any existing proteins in the NCBI non-redundant protein database. Several complete genomes of rV5-like viruses are now available. The viruses share a high number of proteins, but based on whole-genome comparisons, two rV5-like sub-groups may exist, rV5 and *Salmonella* phage PVP-SE1 (10–12). vB_EcoM_FFH_2 has significantly more sequence similarity to rV5 (both of which were isolated using *E. coli* O157:H7), which would make it a member of the rV5 sub-group.

### Nucleotide sequence accession numbers.

The complete sequences of vB_EcoS_FFH_1 and vB_EcoM_FFH_2 were deposited in GenBank under the accession numbers KJ190157 and KJ190158, respectively.
